# A New Recurrence-Network-Based Time Series Analysis Approach for Characterizing System Dynamics

**DOI:** 10.3390/e21010045

**Published:** 2019-01-09

**Authors:** Guangyu Yang, Daolin Xu, Haicheng Zhang

**Affiliations:** State Key Laboratory of Advanced Design and Manufacturing for Vehicle Body, Hunan University, Changsha 410082, China

**Keywords:** complex network, time series analysis, chaos

## Abstract

In this paper, a novel analysis method based on recurrence networks is proposed to characterize the evolution of dynamical systems. Through phase space reconstruction, a time series was transformed into a high-dimensional recurrence network and a corresponding low-dimensional recurrence network, respectively. Then, two appropriate statistics, the correlation coefficient of node degrees (CCND) and the edge similarity, were proposed to unravel the evolution properties of the considered signal. Through the investigation of the time series with distinct dynamics, different patterns in the decline rate of the CCND at different network dimensions were observed. Interestingly, an exponential scaling emerged in the CCND analysis for the chaotic time series. Moreover, it was demonstrated that the edge similarity can further characterize dynamical systems and provide detailed information on the studied time series. A method based on the fluctuation of edge similarities for neighboring edge groups was proposed to determine the number of groups that the edges should be partitioned into. Through the analysis of chaotic series corrupted by noise, it was demonstrated that both the CCND and edge similarity derived from different time series are robust under additive noise. Finally, the application of the proposed method to ventricular time series showed its effectiveness in differentiating healthy subjects from ventricular tachycardia (VT) patients.

## 1. Introduction

In the last few decades, complex network theory [1,2,3,4,5,6,7,8] has undergone vast developments since the first reports of small world networks [9] and scale free networks [10]. The theory of complex networks provides us with a new perspective to understand complex systems. A resulting application field of these theories is the use of complex network methods to identify the characteristics of time series. Zhang and Small [11,12] first attempted the transformation from the pseudo-periodic time series to a complex network, which considered each cycle of a time series as a node in the network. Lacasa et al. [13] proposed a visibility graph method by taking individual observations as nodes and establishing connectivity according to a partial convexity constraint. McCullough et al. and Sun et al. [14,15] proposed ordinal partition networks that divide the phase space of a dynamical system into *K* disjoint sets and transform the evolution of the system into a sequence of symbols. The network construction methods mentioned above have the specific advantage of a low cost in computations because the embedding step can be avoided, but it may be difficult for them to obtain sufficient information from high-dimensional systems.

Takens’ embedding theorem [16] for phase space reconstruction states that, if the time delay and embedding dimension are chosen appropriately, the distribution of state vectors in phase space will reflect the underlying dynamics of the original systems. Donner et al. [17] provided a full classification of networks based on neighborhood relationships between state vectors in the reconstructed phase space of a dynamical system, distinguishing between the *k*-nearest neighbor networks [18], adaptive nearest neighbor networks [19], and epsilon-recurrence networks [20,21,22]. The epsilon-recurrence networks form the class of recurrence networks in the strict sense, according to the most common definition of recurrences in phase space [23]. In this type of time series networks, nodes represent state vectors in the reconstructed phase space, and the distance thresholds that determine the connection between nodes are chosen according to a fixed link density or a fixed phase space distance [20,21,22]. If the distance between two nodes is smaller than or equal to the threshold, two nodes are considered to be connected. The traditional statistical characteristics of recurrence networks only reflect the static properties of a time series, such as the homogeneity of the spatial filling, but the time-ordering information is lost in this framework.

In this paper, a novel method based on epsilon-recurrence networks is proposed for the study of the evolution properties of dynamical systems. For each time series, a high-dimensional recurrence network and a corresponding low-dimensional recurrence network were constructed. The network dimension L represents the number of state vectors that form a node in the network. Specifically, the nodes of the L-dimensional recurrence network are defined by the sequence of L consecutive state vectors, while the nodes of the low-dimensional recurrence network represent single state vectors in phase space. The connection is determined by the distance between the nodes. Two topological statistics, the correlation coefficient of the node degrees and edge similarity, were proposed to characterize the similarity between the high-dimensional recurrence network and the corresponding low-dimensional recurrence network. By analyzing numerical data derived from the Hénon map, lkeda map, folded-towel map, and white noise, the capability of these measures to characterize the dynamic properties of time series in the presence of significant noise was demonstrated. Finally, the application to human electrocardiogram (ECG) recordings showed that network-based statistics can effectively distinguish the cardiac system in both the healthy and ventricular tachycardia (VT) states.

The remainder of this paper is organized as follows: Section 2 introduces the procedures required for the transformation of time series into networks and some topological parameters, which were used to study the similarity between networks; the applications of the proposed method to synthetic data and ECG data are shown in Section 3 and Section 4, respectively; and finally, conclusions are drawn in Section 5.

## 2. Network Construction from Time Series

**Figure 1:**
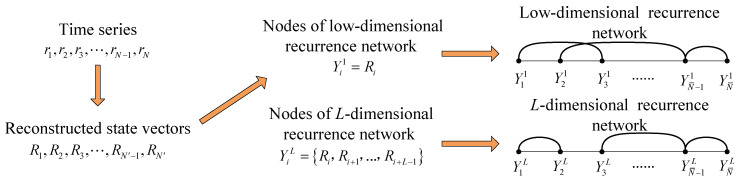
The process of network construction.

We first introduce the procedures required for the construction of complex networks. The process is shown in Figure 1.

Beginning with the phase space reconstruction, according to Takens’ embedding theorem [16], a time delay can be used to reconstruct the phase space from a time series. For a time series with *N* samples, {*r_i_*}, the state vectors in a phase space, can be defined as follows:

(1) $$R_{i}=\left\{r_{i},r_{i+\tau },\ldots ,r_{i+(m-1)\tau }\right\}\;\left(i=1,2,\ldots ,N^{\prime }\right)$$

where τ is the time delay; m is the embedding dimension; and $N^{\prime }$ is the number of reconstructed state vectors, which is equal to N−(m−1)τ.

By reconstructing the phase space from a given time series, a series of state vectors $R_{1},R_{2},\ldots ,R_{k},\ldots ,R_{N^{\prime }}$ can be obtained. Using these state vectors, we can then construct a high-dimensional recurrence network and a corresponding low-dimensional recurrence network, respectively. A node of the L-dimensional recurrence network is defined by the sequence of L consecutive state vectors, for example, $Y_{i}^{L}=\left\{R_{i},R_{i+1},\cdots ,R_{i+L-1}\right\}$ is the *i*-th node in the L-dimensional recurrence network. Each node represents a segment of the phase space trajectory. The distance matrix $D_{L}=(d_{L}^{ij})$ between nodes can be obtained by the equation below:

(2) $$d_{L}^{ij}=\max\left({\left\|R_{i}-R_{j}\right\|}_{\infty },{\left\|R_{i+1}-R_{j+1}\right\|}_{\infty },\ldots ,{\left\|R_{i+L-1}-R_{j+L-1}\right\|}_{\infty }\right)$$

where ${\left\|\;\right\|}_{\infty }$ denotes the maximum norm. For all nodes in the network, this generates a distance matrix, $D_{L}=(d_{L}^{ij})$, which reflects the distance between segments of the phase space trajectories with length L. By choosing a threshold $\Delta_{L}$ for the L-dimensional recurrence network, we obtain the adjacency matrix, $A_{L}=(a_{L}^{ij})$: $a_{L}^{ij}=1$ if $d_{L}^{ij}\leq \Delta_{L}$, while $a_{L}^{ij}=0$ if $d_{L}^{ij}>\Delta_{L}$. The conditions $a_{L}^{ij}=1$ and $a_{L}^{ij}=0$ correspond to connection and disconnection, respectively. The constructed network contains $\bar{N}=N^{\prime }-L+1$ nodes.

Subsequently, a corresponding low-dimensional recurrence network, with the same number of nodes $\bar{N}$ as the L-dimensional network, is constructed. Each node of the low-dimensional recurrence network is defined as a state vector in the reconstructed phase space, for example, $Y_{i}^{1}=R_{i}\;(i=1,2,\ldots ,\bar{N})$ is the *i*-th node in the network. The connection between nodes i and j is determined by their distance, which is defined below:

(3) $$d_{1}^{ij}={\left\|R_{i}-R_{j}\right\|}_{\infty }$$

A threshold $\Delta_{1}$ is set for the low-dimensional recurrence network. The distance matrix, $D_{1}=(d_{1}^{ij})$, can then be converted to an adjacency matrix, $A_{1}=(a_{1}^{ij})$: $a_{1}^{ij}=1$ if $d_{1}^{ij}\leq \Delta_{1}$, while $a_{1}^{ij}=0$ if $d_{1}^{ij}>\Delta_{1}$. Similar to the high-dimensional case, $a_{1}^{ij}=1$ and $a_{1}^{ij}=0$ correspond to connection and disconnection, respectively. To avoid self-connection of nodes, we defined $a_{1}^{ii}=a_{L}^{ii}=0$.

The construction of the network is highly dependent on the threshold, Δ, which should be tailored to specific questions that need to be solved. Several strategies for the selection of the threshold have been proposed. It was suggested that choosing a fixed link density is helpful for the estimation of the dynamical properties in many systems [23,24,25]. Therefore, we determined the threshold for the *L*-dimensional recurrence network, Δ*_L_*, by setting a fixed link density, *ρ_L_*, which is defined as follows:

(4) $$\rho_{L}=\frac{1}{\bar{N}(\bar{N}-1)}\sum_{i,j=1}^{\bar{N}}a_{L}^{ij}$$

According to the procedures for network construction introduced above, it is known that the nodes $Y_{i}^{1}$ in low-dimensional recurrence networks correspond to the first components of the state vectors—represented by the nodes $Y_{i}^{L}$ in high-dimensional recurrence networks. For chaotic (deterministic) systems, the evolution trajectories of two close state vectors remain close together for several time steps, thus the high-dimensional recurrence network and the corresponding low-dimensional network are similar. As for random systems, the evolution trajectories starting from nearby initial conditions diverge rapidly over time, leading to a large difference between the high-dimensional network and corresponding low-dimensional network. Therefore, the similarity between the two networks can reflect the evolution properties of the studied dynamical systems that are captured in a single observational time series. In this study, the link densities of the high-dimensional recurrence network and the corresponding low-dimensional recurrence network were set at the same value, so that the two networks were comparable.

According to the analysis above, it can be seen that the similarity between the high-dimensional recurrence network and the corresponding low-dimensional recurrence network is related to the evolution properties of dynamical systems. A quantitative measure is thus proposed to characterize the similarity between networks by considering the correlation of node degrees. The degree of node i in an L-dimensional recurrence network represents the number of nodes that are directly connected with i, as represented below:

(5) $$E_{L}^{i}=\sum_{j=1}^{\bar{N}}a_{L}^{ij}$$

The correlation coefficient of the node degree between an L-dimensional recurrence network and the corresponding low-dimensional recurrence network is defined as follows:

(6) $$\mathrm{CCND}=\frac{\sum_{i=1}^{\bar{N}}\left[E_{L}^{i}-\langle E_{L}\rangle ]\cdot [E_{1}^{i}-\langle E_{1}\rangle \right]}{\sqrt{\sum_{i=1}^{\bar{N}}{\left[E_{L}^{i}-\langle E_{L}\rangle \right]}^{2}}\cdot \sqrt{\sum_{i=1}^{\bar{N}}{\left[E_{1}^{i}-\langle E_{1}\rangle \right]}^{2}}}$$

where $\langle E_{L}\rangle =\sum_{i=1}^{\bar{N}}E_{L}^{i}/\bar{N}$ and $\langle E_{1}\rangle =\sum_{i=1}^{\bar{N}}E_{1}^{i}/\bar{N}$ represent the average node degrees of an L-dimensional recurrence network and the corresponding low-dimensional recurrence network, respectively. The correlation measure correlation coefficient of node degrees (CCND) is restricted to the range [−1,1] and CCND = 1, 0, and −1 represent perfect correlation, no correlation, and perfect anti-correlation, respectively. It should be noted that in a low-dimensional recurrence network (i.e., $Y_{i}^{1}=R_{i}\;(i=1,2,\ldots ,\bar{N})$), only the first $\bar{N}$ nodes are considered. This is because of the fact that the main purpose of Equation (6) is to study to what extent the node degrees of an L-dimensional recurrence network are related to their initial states (corresponding low-dimensional recurrence network) after L−1 time steps. Because of the finite length of the studied time series, there were no corresponding nodes in L-dimensional recurrence networks for nodes $Y_{i}^{1}\;(i>\bar{N})$, therefore, these nodes could be neglected.

The degree of a node in low-dimensional recurrence networks reflects the local phase space density, while the degree of a node in high-dimensional recurrence networks represents the density of state vectors in the high-dimensional phase space. For a deterministic system, two close state vectors remain close together during the evolution for several time steps, so the node degrees of high-dimensional recurrence networks are strongly correlated with those of the corresponding low-dimensional recurrence networks. However, if the system is uncorrelated or weakly correlated, the evolution trajectories are random and unpredictable. This results in a smaller correlation of node degrees between the high-dimensional recurrence network and the corresponding low-dimensional recurrence network. Therefore, the CCND can characterize the evolution properties of dynamical systems.

Another measure called the edge similarity is proposed to study the relationship between the distance of the state vectors and the similarity of their evolution trajectories, which cannot be characterized by CCND. For an edge between two nodes i and j in the low-dimensional recurrence network, a weight, $w_{ij}$, is assigned according to its distance, $d_{1}^{ij}$. The weighted edges are partitioned into n groups according to a mapping rule, f, and each group is labeled by an index, I. The mapping rule, f, is defined as follows:

(7) $$f(w_{ij})=\{\begin{array}{ll} 1 & 0\leq (w_{ij}-\min(w_{ij}))/w\leq 1\\ \cdots & \cdots \\ I & I-1<(w_{ij}-\min(w_{ij}))/w\leq I\\ \cdots & \cdots \\ n & n-1<(w_{ij}-\min(w_{ij}))/w\leq n \end{array}$$

where $w=(\max(w_{ij})-\min(w_{ij}))/n$ indicates the size of each group. The index, I, reflects the values of edge weights that belong to that group (i.e., an edge with a smaller weight is assigned to a group with a smaller index and vice versa). The edge similarity between the L-dimensional recurrence network and the low-dimensional recurrence network in the *I*-th group is then defined as follows:

(8) $$\eta \left(I\right)=\frac{C\left(I\right)}{N\left(I\right)}$$

where N(I) is the number of edges that belong to the *I*-th group and C(I) is the number of edges that satisfy $a_{1}^{ij}=a_{L}^{ij}=1$ in the *I*-th group. Thus, the edge similarity, η(I), represents the probability that an edge in the low-dimensional recurrence network will also remain in the high-dimensional recurrence network within group I. The dynamic properties of time series are characterized by the differences of edge similarities between different groups.

## 3. Analysis of Synthetic Data

We first illustrated the potential of the proposed method by several typical dynamic systems; namely, the (1) Hénon map, (2) lkeda map, (3) folded-towel map, and (4) white noise.

The Hénon map [26] is a discrete-time dynamical system. It is one of the examples of dynamical systems that exhibit chaotic behavior, which has been studied the most. The Hénon map takes a point $\left(x_{i},y_{i}\right)$ in the plane and maps it to a new point, as shown below:

(9) $$\{\begin{array}{l} x_{i+1}=1-\bar{a}x_{i}^{2}+y_{i}\\ y_{i+1}=\bar{b}x_{i} \end{array}$$

The map depends on two parameters $\bar{a}$ and $\bar{b}$. We chose $\bar{a}=1.43$ and $\bar{b}=0.3$, which determined that the Hénon map is chaotic.

The lkeda map was first proposed by lkeda [27] as a model of light going around a nonlinear optical resonator, which is defined as follows:

(10) $$\{\begin{array}{l} x_{i+1}=1+\mu (x_{i}\cos t_{i}-y_{i}\sin t_{i})\\ y_{i+1}=\mu (x_{i}\sin t_{i}+y_{i}\cos t_{i}) \end{array}$$

where μ is a parameter and $t_{i}=0.4-6/(1+x_{i}^{2}+y_{i}^{2})$. For μ≥0.6, this system has a chaotic attractor. In this study, we chose μ=0.7.

We also considered the following folded-towel map, introduced by Rössler [28]:

(11) $$\{\begin{array}{l} x_{i+1}=\bar{a}x_{i}(1-x_{i})-0.05(y_{i}+0.35)(1-2z_{i})\\ y_{i+1}=0.1((y_{i}+0.35)(1+2z_{i})-1)(1-1.9x_{i})\\ z_{i+1}=3.78z_{i}(1-z_{i})+\bar{b}y_{i} \end{array}$$

For $\bar{a}=1.9$ and $\bar{b}=0.2$, this map is hyper-chaotic with two positive Lyapunov exponents, so it can generate more complex dynamics than the Hénon map and lkeda map.

We applied the proposed method to time series from different models and characterized the system dynamics by the network-based measures proposed in the last section. All the data were derived from the *x* components of the chaotic maps. For each case, we computed a time series of length 6000 and the first 1000 data points were removed in order to exclude transient dynamics. Moreover, the mutual information method [29] was used to determine the time delay *τ* and a large embedding dimension m=5 was chosen to reliably unfold the fine structure [19]. It should be noted that the mutual information method cannot be directly applied to the discrete chaotic time series. This is because the sampling interval of the discrete chaotic time series is large, which causes rapid variations in the mutual information with respect to τ. The resulting curves of the mutual information calculated from the discrete chaotic series are thus similar to the curves from the white noise series. Thus, the discrete chaotic time series should be interpolated before determining its time delay. For any two adjacent data points, T data points were interpolated with a spline function in this study. The data and the reconstructed state vectors after interpolation can be represented as $\left\{{\bar{r}}_{i}\right\}\;\left(i=1,2,\ldots ,M\right)$ and ${\bar{R}}_{i}=\left\{{\bar{r}}_{i},\;{\bar{r}}_{i+\tau },\;\ldots ,\;{\bar{r}}_{i+(m-1)\tau }\right\}\;\left(i=1,2,\ldots ,M^{\prime }\right)$, respectively, where M=(N−1)T+N and $M^{\prime }=M-(m-1)\tau$. Thus, each node of an L-dimensional recurrence network is formed by $L^{\prime }=(L-1)T+L$ consecutive state vectors, which can be represented as ${\bar{Y}}_{i}^{L}=\left\{{\bar{R}}_{i},\;{\bar{R}}_{i+1},\ldots ,{\bar{R}}_{i+L^{\prime }-1}\right\}\;\left(i=1,2,\ldots ,\bar{M}\right)$, where $\bar{M}=M^{\prime }-L^{\prime }+1$. In this study, we chose T=9. As the white noise series has no attractor structure, its time delay was set to 1, while for other dynamical systems, the time delay was determined by the mutual information method [29].

**Figure 2:**
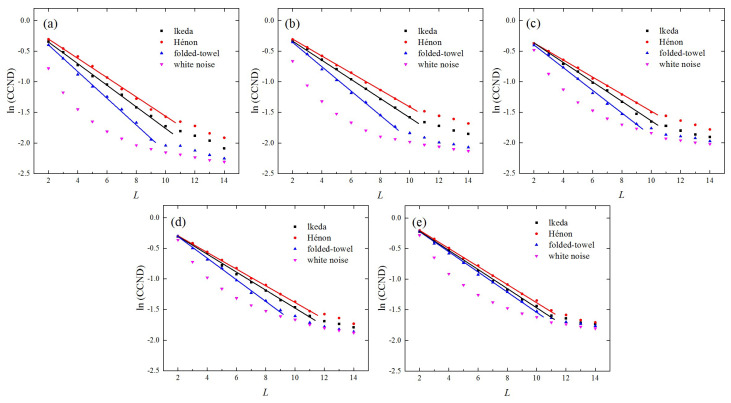
Variations of the correlation coefficient of node degrees (CCND), as a function of the network dimension L, resulting from the lkeda map, Hénon map, folded-towel map, and white noise series. (**a**) ρ=0.05; (**b**) ρ=0.1; (**c**) ρ=0.2; (**d**) ρ=0.3; (**e**) ρ=0.4.

According to previous studies [25,30], a small distance threshold may be preferable for the construction of recurrence networks from a time series. Because a large threshold may obscure or conceal the local characteristics by over-connecting the nodes, we began the analysis with a relatively small link density by setting ρ to 0.05. Figure 2a shows the CCND as a function of the network dimension L. The CCNDs derived from different systems all decreased with the increase of L. This indicates that higher-dimensional recurrence networks are less correlated with the corresponding low-dimensional recurrence networks. Moreover, the CCNDs generated from different types of time series exhibited distinct decreasing patterns. For chaotic series, an exponential behavior was observed between the CCND and L (i.e., $\mathrm{CCND}\propto e^{-\gamma L}$). The parameter γ is an exponential scaling factor, which indicates the rate of decrease of the CCND. It was found that the scaling factor, γ, resulting from the folded-towel map, was larger than those from the lkeda map and the Hénon map. In comparison, the CCND resulting from white noise series was smaller than that of the chaotic series, especially for small values of L, and no exponential relationship was observed.

These observations indicate that the dynamic properties of the system under study play an important role in shaping the corresponding network properties. The findings can be explained by the evolutionary processes of different systems. One possible reason for the exponential decrease of the CCND is its dependence on the variation in the number of nearby trajectories of nodes over time. For chaotic systems, it is largely dominated by the characteristic of the exponential divergence of nearby state vectors, so the CCND derived from chaotic series also shows a similar exponential behavior. However, the reasoning is heuristic and not yet supported by some deep theories, which needs further research in the future. The semi-logarithmic plot ln(CCND)∝L can be observed as being approximate to a straight line, with a slope that exhibits a statistical relationship with the largest Lyapunov exponent, λ. The largest Lyapunov exponents of time series derived from the lkeda map, Hénon map, and folded-towel map were 0.453, 0.418, and 0.693, respectively, which were calculated by the Time Series Analysis (TISEAN) software (TISEAN 2.1) package [31]. It is clear that a chaotic series with larger λ results in a steeper slope of the CCND because the chaotic series with a larger λ is less regular and less predictable, leading to a rapid decrease in the correlation of node degrees between a high-dimensional recurrence network and the corresponding low-dimensional recurrence network. As for white noise series, the process is completely stochastic and unrelated to its previous state. The irregularity and unpredictability imply that there is no statistical relationship between the distance of the two state vectors and the similarity of their evolution trajectories. Hence, the CCND resulting from the white noise series decreased rapidly with the increase of L. It should be noted that the CCNDs derived from different time series tended to saturate for the high-dimensional recurrence network. This is because higher-dimensional recurrence networks change fewer and fewer of the distance values between the nodes [24], which was also observed in numerical investigations associated with the false nearest neighbor method [32]. In addition, the saturation behavior observed was also partially the result of the finite sample size.

We then considered the choice of the link density in order to make sure that the constructed networks can truly represent the characteristics of dynamical systems. To study this, we further calculated the CCNDs from different systems by varying ρ from 0.1 to 0.4, as shown in Figure 2b–e. It was found that for ρ=0.1, the differences between the curves of CCNDs from various time series were large, which was similar to the case of ρ=0.05. This indicates that the results are robust for a relatively small ρ. However, if ρ continues to increase, the curves of CCNDs for different series will approach each other and gradually become difficult to distinguish. This is because, with the increase of ρ, many nodes that are far away from each other are connected. This results in obscuration or concealment of the evolution properties of dynamical systems [33]. Therefore, a value of link density ρ that does not exceed 0.1 is preferable for a feasible analysis of the constructed network.

**Figure 3:**
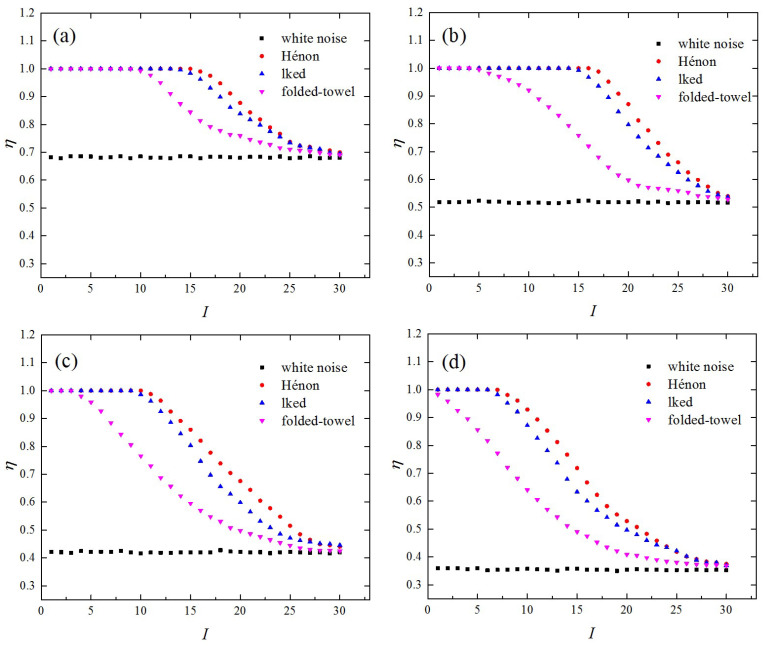
The edge similarities of different I resulting from the lkeda map, Hénon map, folded-towel map, and white noise series with ρ=0.1 and n=30. (**a**) L=2; (**b**) L=3; (**c**) L=4; (**d**) L=5.

The effectiveness of the CCND analysis for the evaluation of the evolution properties of dynamical systems was confirmed. We also illustrated that the edge similarity introduced in Section 2 can further characterize different time series. In Figure 3a, we show the edge similarities of different I with network dimension L=2, link density ρ=0.1, and number of groups n=30. The edge similarities, η, derived from the lkeda map and the Hénon map remained around 1 until I=15 and then decreased rapidly with I, while the edge similarity, η, resulting from the folded-towel map remained at around 1 for I<11 and decreased at smaller I (I=11). As for white noise series, the edge similarity, η, was independent of I and remained stable at around 0.681. These interesting results are relevant to the evolutionary processes of different dynamical systems. It is well known that a chaotic map is a deterministic system, with the nature of sensitivity to initial conditions. The short-term evolution of trajectories starting from nearby initial conditions is strongly correlated. Thus, for chaotic systems, close state vectors remain close together for several time steps and the edge similarities for small I are large. In comparison, the trajectories evolving from far away state vectors will decrease in similarity, resulting in small edge similarities η for large I. The edge similarities, η, resulting from the folded-towel map were obviously smaller than those from the lkeda and the Hénon maps for I∈(11,30), because the time series derived from the folded-towel map was more chaotic and unpredictable. As for the white noise series, the evolution was totally random and independent of the previous states, so the edge similarities of different groups were similar.

From the above analysis, it can be seen that the evolution properties of different dynamical systems can be well characterized by the edge similarity. Moreover, the effect of the network dimension, L, on the edge similarity was further evaluated. The edge similarities of different I resulting from various time series with L=3,4,5 are shown in Figure 3b–d, respectively. The edge similarity was found to decrease with an increase of the network dimension, L, because close state vectors diverge quickly over time as a result of the randomness of the studied dynamical systems. For chaotic series, the edge similarities of smaller I remained relatively large for higher-dimensional recurrence networks, because state vectors with smaller distances were more similar during the evolutionary process and their trajectories remained close for a longer period of time. As for white noise series, the edge similarities of different groups decreased to a similar extent with the increase of L. The distance threshold $\Delta_{L}$ of the L-dimensional recurrence networks from white noise series corresponding to $\rho_{L}$ is equal to the maximum distance of the nearest $\tilde{N}$ pairs of sampling data, where $\tilde{N}=\left[\sqrt[{\left(L+m-1\right)}]{\rho_{L}}N(N-1)\right]$ and [x] denotes the largest integer no more than x. The edge similarity of white noise series for the network dimension, L, can then be represented as $\eta ={\left(\sqrt[{\left(L+m-1\right)}]{\rho_{L}}\right)}^{L-1}$. Thus, for L=2,3,4,5, the edge similarities resulting from the white noise series were around 0.681, 0.518, 0.422, and 0.359, respectively. According to Figure 3, the distinctions of edge similarities between chaotic time series and white noise time series were maintained when L changed from 2 to 5.

It should be noted that the results shown above were dependent of the number of groups, n, so the choice of this parameter needs to be studied carefully. As introduced in Section 2, the edge similarity was proposed to study the relationship between the distances of the connected state vectors and the similarity of their evolution trajectories. The aim of the grouping is a simplification of the distances between connected state vectors. It is clear that increasing the number of groups, n, leads to the increase of the resolution of edge weights (i.e., the distances between connected state vectors). However, because of the finite sample size, the number of edges in each group deceases in accordance with the increase of $\Delta_{1}$, which may cause a large statistical fluctuation in the edge similarity. Considering that the edges in adjacent groups have similar weights, the edge similarities of adjacent groups were also supposed to be similar. In this study, we defined the maximum fluctuation (MF) of edge similarities for neighboring edge groups as follows:

(12) MF(n)=max(|η(1)−η(2)|,|η(2)−η(3)|,…,|η(n−1)−η(n)|)

**Figure 4:**
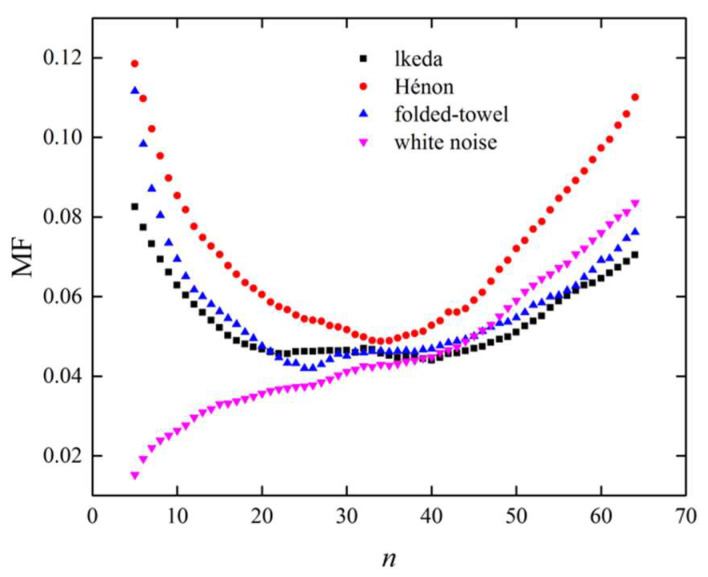
Variations in maximum fluctuations (MFs) derived from different time series, as a function of n with sample length N=5000, network dimension L=2, and link density ρ=0.1.

The choice of n should make the value of MF small, which indicates that there is a higher resolution of the edge weights and a small statistical fluctuation in edge similarities. Figure 4 shows the MFs derived from different time series as a function of n with sample length N=5000, network dimension L=2, and link density ρ=0.1. For the chaotic series, the MFs decreased rapidly when n increased from 5 to 20. This is because the edges in adjacent groups tend to have greater similarity in weight with the increase of n, leading to a smaller difference of edge similarities between adjacent groups. In the range 20≤n≤40, the MFs resulting from chaotic series were stable at a relatively small value. The small difference of edge similarities between adjacent groups indicates that the resolution of edge weights was relatively high. However, when continuing to increase n, the MFs increased. This is because a large n causes a decrease in the number of edges in each group, which leads to increased statistical fluctuations. As for the white noise series, the MF increased monotonously with n. This is because the edge similarities in different groups are similar and increasing n simply leads to fewer edges in each group, which results in larger statistical fluctuations. According to the results shown in Figure 4, it was concluded that the number of groups, n, should be set at [20,40]—where the MFs derived from different time series exhibited relatively small values.

**Figure 5:**
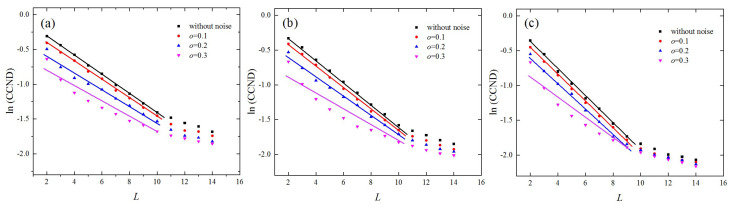
Variations in the CCNDs as a function of network dimension, L, resulting from chaotic series contaminated by noise of different levels where ρ=0.1. The (**a**) Hénon map; (**b**) lkeda map; and (**c**) folded-towel map.

Because empirical data were contaminated by noise, in this section, we also studied the robustness of the proposed method against noise by analyzing the chaotic series corrupted by white noise. The corrupted signal, s, was formed by the composition of the normalized time series, x (μ=0,σ=1), and Gaussian white noise, ξ, i.e., s=x+oξ, where μ is the mean, σ is the standard deviation, and o is the noise level. We numerically studied the variations in the CCND with L under different noise levels, o, by fixing ρ=0.1. The results for the lkeda, Hénon, and folded-towel maps are shown in Figure 5a–c, respectively. When the noise level, o, was lower than 0.1, the variations in the CCND as a function of L were similar to those derived from the time series without noise and the exponential behaviors between the CCND and L were maintained when the noise level, o, was less than 0.2. However, when the noise level increased to 0.3, the corresponding topological structures of networks were distorted from the original series and the exponential relationships were weakened. This is because high levels of noise obscure the dynamic characteristics of chaotic systems, which causes the decline patterns of CCND derived from the chaotic series to approach that obtained from the white noise.

**Figure 6:**
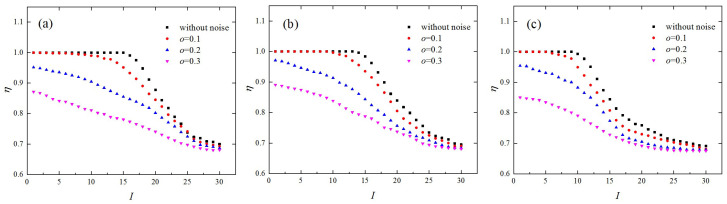
The edge similarities resulting from chaotic time series contaminated by the noise of different levels, where ρ=0.1 and L=2. The (**a**) Hénon map; (**b**) lkeda map; and (**c**) folded-towel map.

The influence of noise on the edge similarity was also evaluated. Figure 6 depicts the edge similarities derived from different chaotic maps in the presence of various levels of noise, where the network dimension was set as L=2 and the link density was set as ρ=0.1. With the increase of o, edge similarities in different groups decreased, especially for small values of I. The edge similarities generated from the lkeda map and the Hénon map without noise were about 1 for I<16, but they decreased to below 0.9 when the noise level, o, increased to 0.3 (Figure 6a,b). As for the folded-towel map, the edge similarities for I<11 had large values when o=0 and decreased to less than 0.86 when the noise level, o, increased to 0.3 (Figure 6c). This was because two state vectors that are nearby in phase space may become distant from each other for the next time step because of the influence of Gaussian white noise. However, there remains an obvious dependence of the edge similarity, η, on the group index, I, in the presence of noise with level o=0.3, which is distinct from a white noise series.

**Figure 7:**
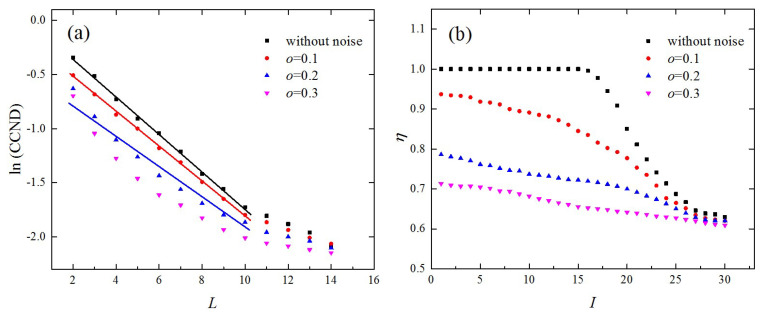
Results for the proposed topological statistics derived from the time series of the lkeda map contaminated by the noise of different levels; (**a**) variations of the CCND, as a function of the network dimension L, and (**b**) edge similarity.

We also studied the robustness of the CCND and edge similarity against noise with ρ=0.05. The time series from the lkeda map was used as an example and the results are shown in Figure 7. According to Figure 7a, although the exponential behavior between the CCND and L was maintained for noise level o=0.1, the variations in the CCND as a function of L deviated obviously from those without noise. When o further increased to 0.2, the exponential relationship weakened and no exponential behavior was observed for o=0.3. The edge similarity, η, as shown in Figure 7b, decreased significantly with the increase of o. When o increased to 0.3, the edge similarities were small even for small values of I. From Figure 5b, Figure 6b, and Figure 7, it can be seen that both the CCND and edge similarity with ρ=0.05 were more sensitive to noise compared with those of ρ=0.1. This is because when choosing a small link density, the connections between nodes are more easily affected by noise. Therefore, the proposed topological statistics are more robust under additive noise when the link density, ρ, is set to 0.1.

## 4. Analysis of Human ECG Data

**Figure 8:**
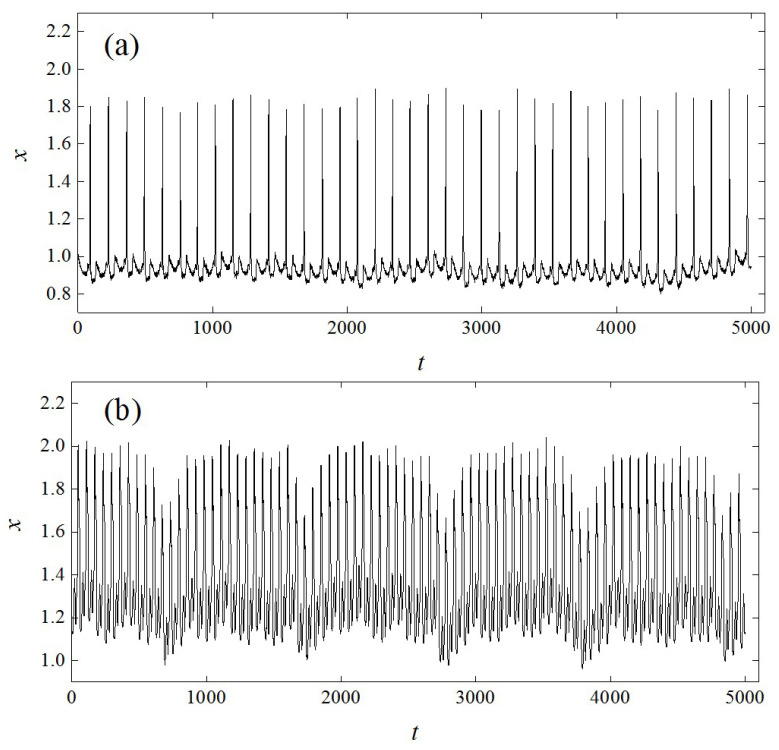
Two representative recordings used in this study. (**a**) Normal sinus rhythm (NSR); (**b**) ventricular tachycardia (VT).

**Figure 9:**
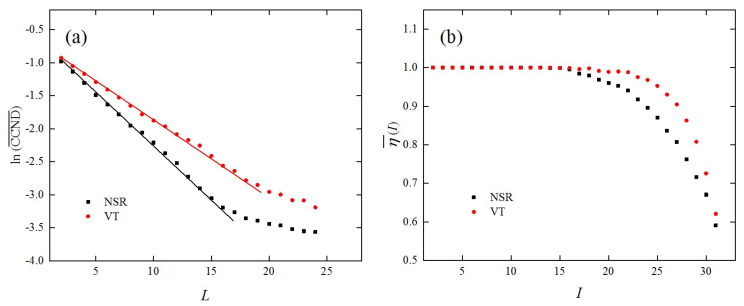
The network-based measures derived from the NSR and the VT recordings. (**a**) The average CCNDs as a function of the network dimension L, with ρ=0.1; (**b**) the average edge similarities of different I, with L=2, n=30, and ρ=0.1.

Finally, the proposed method was applied to the human ECG based on the Massachusetts Institute of Technology human heartbeat database, which is a large archive of well-characterized digital recordings of physiological signals, time series, and related data for use by the biomedical research community. Two well-studied cases were considered, namely normal sinus rhythm (NSR) data and ventricular tachycardia data (Figure 8) in the database [34], to illustrate the effectiveness of the proposed method. The database has a total of 35 subjects and for each subject, 127,000 data points were recorded. We randomly selected 50 NSR episodes and 50 VT episodes from all 35 subjects and each episode contained 5000 data points. The embedding dimension, m, and time delay, τ, were determined by the false nearest neighbors method [32] and the mutual information method [29], respectively. The CCNDs of all 100 episodes were calculated under different network dimensions, L. The average CCNDs as a function of L for both the NSR data and the VT data are shown in Figure 9a. The results show that the CCND decreased exponentially with the network dimension, L, which is consistent with the qualitative behavior of chaotic systems. This finding indicates that human cardiac signals have chaotic properties, which is consistent with several previous studies [11,35]. Moreover, it should be noted that the scaling factors, *γ*, were different for the two types of ECG recordings. For VT, the cardiac system presented a more orderly and predictable behavior resulting in a smaller scaling factor, γ, while the NSR signal was more chaotic and unpredictable, which led to a rapid decrease of CCND with the increase of L. Moreover, the decay rates of the CCND derived from both the NSR and the VT recordings decreased for larger network dimensions L. This was related to the expected saturation of distances between nodes as mentioned above.

The average edge similarities for different I for the NSR data and the VT data also showed some differences (Figure 9b). Specifically, the edge similarities, η, were stable and remained around 1 for VT when I≤23 and dropped rapidly for I>23. In comparison, the edge similarities for the NSR signals were relatively large until I=16 and became smaller than those for the VT signals for I>16. This is because the cardiac system tends to be more uncorrelated in the healthy state [36]. Based on the results above, it was concluded that the proposed method can be used to classify and characterize the human ECG data from a novel perspective.

## 5. Conclusions

In summary, a simple and straightforward analysis method was proposed for recurrence networks derived from time series, which can characterize the evolution properties of dynamical systems. For each time series, a high-dimensional recurrence network and a corresponding low-dimensional recurrence network were constructed. The CCND was proposed to study the correlation of node degrees between the networks. It was found that calculating the CCND for time series can lead to the effective distinction between various types of time series. Specifically, for chaotic series, there was an exponential relationship between the CCND and the network dimension, L, while no exponential relationship was observed for the white noise series. Moreover, the edge similarity between high-dimensional recurrence networks and corresponding low-dimensional recurrence networks was studied. Each edge in the low-dimensional recurrence network was weighted according to its distance and the edges were partitioned into n groups based on their weights. For chaotic time series, the edge similarities were found to be dependent on the group index, I. In comparison, the edge similarities resulting from white noise series had small values for all groups. A method based on the fluctuation of edge similarities for neighboring edge groups was proposed to determine the number of groups that edges should be partitioned into. In addition, we tested the proposed topological statistics by analyzing chaotic series contaminated by noise. The results revealed that our method exhibits good robustness against noise. Finally, the application of the proposed method to human electrocardiograms demonstrated its effectiveness in differentiating between healthy subjects and VT patients.

## References

[1] Jeong H., Mason S.P., Barabási A.L., Oltvai Z.N. (2001). Lethality and centrality in protein networks. Nature.

[2] Nishikawa T., Motter A.E., Lai Y.C., Hoppensteadt F.C. (2003). Heterogeneity in oscillator networks: are smaller worlds easier to synchronize?. Phys. Rev. Lett..

[3] Kim J.S., Goh K.I., Kahng B., Kim D. (2007). A box-covering algorithm for fractal scaling in scale-free networks. Chaos.

[4] Sprott J.C. (2008). Chaotic dynamics on large networks. Chaos An Interdiscip. J. Nonlinear Sci..

[5] Ma X., Huang L., Lai Y.C., Wang Y., Zheng Z. (2008). Synchronization-based scalability of complex clustered networks. Chaos.

[6] Rosvall M., Esquivel A.V., Lancichinetti A., West J.D., Lambiotte R. (2014). Memory in network flows and its effects on spreading dynamics and community detection. Nat. Commun..

[7] Hou L., Small M., Lao S. (2015). Dynamical Systems Induced on Networks Constructed from Time Series. Entropy.

[8] Zufiria P.J., Barriales-Valbuena I. (2017). Entropy Characterization of Random Network Models. Entropy.

[9] Watts D.J., Strogatz S.H. (1998). Collective dynamics of “small-world”networks. Nature.

[10] Barabasi A.L., Albert R. (1999). Emergence of scaling in random networks. Science.

[11] Zhang J., Small M. (2006). Complex network from pseudoperiodic time series: topology versus dynamics. Phys. Rev. Lett..

[12] Zhang J., Sun J., Luo X., Zhang K., Nakamura T., Small M. (2008). Characterizing pseudoperiodic time series through the complex network approach. Phys. D Nonlinear Phenom..

[13] Lacasa L., Luque B., Ballesteros F. (2008). From time series to complex networks: the visibility graph. Proc. Natl. Acad. Sci. USA.

[14] McCullough M., Small M., Stemler T., Iu H.H.-C. (2015). Time lagged ordinal partition networks for capturing dynamics of continuous dynamical systems. Chaos An Interdiscip. J. Nonlinear Sci..

[15] Sun X., Small M., Zhao Y., Xue X. (2014). Characterizing system dynamics with a weighted and directed network constructed from time series data. Chaos An Interdiscip. J. Nonlinear Sci..

[16] Takens F. (1981). Detecting Strange Attractors in Turbulence. Dynamical Systems and Turbulence Lecture.

[17] Donner R.V., Small M., Donges J.F., Marwan N., Zou Y., Xiang R., Kurths J. (2011). Recurrence-based time series analysis by means of complex network methods. Int. J. Bifurc. Chaos.

[18] Shimada Y., Kimura T., Ikeguchi T. (2008). Analysis of Chaotic Dynamics Using Measures of the Complex Network Theory. Artificial Neural Networks–ICANN 2008.

[19] Xu X., Zhang J., Small M. (2008). Superfamily phenomena and motifs of networks induced from time series. Proc. Natl. Acad. Sci. USA.

[20] Marwan N., Donges J.F., Zou Y., Donner R.V., Kurths J. (2009). Complex network approach for recurrence analysis of time series. Phys. Lett. A.

[21] Donner R.V., Zou Y., Donges J.F., Marwan N., Kurths J. (2010). Recurrence networks–A novel paradigm for nonlinear time series analysis. New J. Phys..

[22] Gao Z., Jin N. (2009). Complex network from time series based on phase space reconstruction. Chaos An Interdiscip. J. Nonlinear Sci..

[23] Marwan N., Romano M.C., Thiel M., Kurths J. (2007). Recurrence plots for the analysis of complex systems. Phys. Rep..

[24] Kraemer K.H., Donner R.V., Heitzig J., Marwan N. (2018). Recurrence threshold selection for obtaining robust recurrence characteristics in different embedding dimensions. Chaos An Interdiscip. J. Nonlinear Sci..

[25] Schinkel S., Dimigen O., Marwan N. (2008). Selection of recurrence threshold for signal detection. Eur. Phys. J. Spec. Top..

[26] Hénon M. (1976). A two-dimensional mapping with a strange attractor. The Theory of Chaotic Attractors.

[27] Hammel S.M., Jones C., Moloney J.V. (1985). Global dynamical behavior of the optical field in a ring cavity. JOSA B.

[28] Rossler O.E. (1979). An equation for hyperchaos. Phys. Lett. A.

[29] Fraser A.M., Swinney H.L. (1986). Independent coordinates for strange attractors from mutual information. Phys. Rev. A.

[30] Donner R.V., Zou Y., Donges J.F., Marwan N., Kurths J. (2010). Ambiguities in recurrence-based complex network representations of time series. Phys. Rev. E Stat. Nonlinear Soft Matter Phys..

[31] Hegger R., Kantz H., Schreiber T. (1999). Practical implementation of nonlinear time series methods: The TISEAN package. Chaos An Interdiscip. J. Nonlinear Sci..

[32] Kennel M.B., Brown R., Abarbanel H.D. (1992). Determining embedding dimension for phase-space reconstruction using a geometrical construction. Phys. Rev. A At. Mol. Opt. Phys..

[33] Jacob R., Harikrishnan K.P., Misra R., Ambika G. (2016). Can recurrence networks show small-world property?. Phys. Lett. A.

[34] Creighton University Ventricular Tachyarrhythmia Database. http://www.physionet.org/physiobank/database/cudb/.

[35] Small M., Yu D., Harrison R.G. (2001). Surrogate test for pseudoperiodic time series data. Phys. Rev. Lett..

[36] Small M., Yu D., Simonotto J., Harrison R.G., Grubb N., Fox K.A.A. (2002). Uncovering non-linear structure in human ECG recordings. Chaos Solitons Fractals.

